# Photodeposited Pd Nanoparticles with Disordered Structure for Phenylacetylene Semihydrogenation

**DOI:** 10.1038/srep42172

**Published:** 2017-02-08

**Authors:** Qining Fan, Sha He, Lin Hao, Xin Liu, Yue Zhu, Sailong Xu, Fazhi Zhang

**Affiliations:** 1 State Key Laboratory of Chemical Resource Engineering, Beijing University of Chemical Technology, Beijing 100029, China

## Abstract

Developing effective heterogeneous metal catalysts with high selectivity and satisfactory activity for chemoselective hydrogenation of alkyne to alkene is of great importance in the chemical industry. Herein, we report our efforts to fabricate TiO_2_-supported Pd catalysts by a photodeposition method at room temperature for phenylacetylene semihydrogenation to styrene. The resulting Pd/TiO_2_ catalyst, possessing smaller Pd ensembles with ambiguous lattice fringes and more low coordination Pd sites, exhibits higher styrene selectivity compared to two contrastive Pd/TiO_2_ samples with larger ensembles and well-organized crystal structure fabricated by deposition-precipitation or photodeposition with subsequent thermal treatment at 300 °C. The sample derived from photodeposition exhibits greatly slow styrene hydrogenation in kinetic evaluation because the disordered structure of Pd particles in photodeposited Pd/TiO_2_ may prevent the formation of β-hydride phases and probably produce more surface H atoms, which may favor high styrene selectivity.

The chemoselective hydrogenation of alkyne, a hydrocarbon with a carbon-carbon triple bond, is of fundamental importance for the production of fine chemicals and industrial polymerization processes^1^,^2^,^3^. For instance, the removal of phenylacetylene from styrene feeds is crucial in the olefin industry as phenylacetylene acts not only as a kind of impurities in the styrene feed, but also a poison for subsequent polymerization of the styrene. Due to the strict standard for the styrene monomer purity for synthesis of polystyrene, the level of phenylacetylene should be reduced to <10 ppm. The alkyne can be hydrogenated partially to the alkene or fully to the alkane. The most widely used method to remove phenylacetylene is to convert it to styrene through the catalytic semihydrogenation which gives rise to the double benefit of lowering the concentration of phenylacetylene while increasing the production of the desired styrene^4^,^5^,^6^,^7^, in which palladium-based catalysts have received considerable attention owing to their good hydrogenation selectivity toward styrene^7^,^8^. Significant efforts have been devoted to development of novel strategies for the fabrication of highly selective Pd-based catalysts for alkyne hydrogenation. For example, selective deposition of metals/metal oxides, such as Cu^9^, Ag^10^, Si^11^, and TiO_2_^12^,^13^ on the surface of Pd particles promotes the catalytic selectivity *via* geometric and/or electronic effects; Formation of Pd-Cu single atom alloys catalysts enhances the alkene selectivity by tuning the chemical and adsorption properties^14^,^15^; Adopting of Pd-Ga intermetallic compounds with isolation of active Pd sites in the crystallographic structure can obviously improve the hydrogenation selectivity^16^,^17^; Carbon deposited on top Pd layers strongly affects the transport of hydrogen and disturbs the equilibrium of H between surfaces and deeper layers, leading to improved alkene selectivity^18^. Despite these encouraging results, the demand for efficient Pd catalysts with high selectivity to avoid the total hydrogenation of alkyne to alkane still remains.

Various studies have shown that the structure and surface characteristics of Pd metal play important roles in the selective hydrogenation of alkyne to alkene^7^,^8^. Unselective hydrogenation to alkane proceeds on hydrogen-saturated β-hydride (subsurface H strongly coordinates with the Pd atoms in crystal lattices), thus the population of subsurface sites of Pd (the few layers below the surface) had been proposed to govern the hydrogenation events on the metal surface^18^,^19^. Based on temperature-programmed desorption experiments, Khan *et al*.^20^ demonstrated that subsurface H in Pd metal strongly enhances total hydrogenation of acetylene, whereas surface H alone (without any subsurface populations) is much more selective toward ethylene. Thus hindering the participation of subsurface H in the catalytic hydrogenation process by reducing generation of β-hydride phases apparently favors the partial hydrogenation. Herein, the TiO_2_-supported Pd catalyst with unique structural and morphological features of Pd metal particles was fabricated by a photodeposition method to obtain high hydrogenation selectivity of alkyne. The principle of the photodeposition method is that the irradiation of semiconductor substrates with UV light can result in the photoinduced electrons that could reduce the absorbed metal ions to form metal nanoparticles^21^,^22^,^23^,^24^. Several groups^25^,^26^,^27^,^28^,^29^ have made great efforts to investigate the formation mechanism of metal particles on TiO_2_ during photodeposition. From these investigations, it can be revealed that the group VIII noble metals (such as Rh and Pt) can strongly interact with, and are modified by their interaction with, the TiO_2_ support. The procedures of calcination and reduction are omitted for the photodeposition of metal nanoparticles, and the synthesis condition is relatively milder. Therefore, the intrinsic surface structure and electronic properties of the as-synthesized metal catalysts can be sustained for the catalytic reaction. The metal-modified semiconductor samples prepared by this method were often used as photocatalysts showing higher photocatalytic activity than that prepared by other conventional methods. A very recently published contribution reported the synthesizing atomically dispersed Pd catalysts on ultrathin TiO_2_ nanosheets by this photodeposition method, which are active for hydrogenation of C=C and C=O^30^.

In this manuscript, Pd metal nanoparticles were photodeposited on a commercial TiO_2_ support. For comparison, the conventional deposition-precipitation method was used to prepare Pd/TiO_2_ catalysts. The effects of thermal treatment on the structural and morphological characteristics of the photodeposited Pd/TiO_2_ were also investigated. The resulting Pd/TiO_2_ samples were characterized by X-ray diffraction (XRD), inductively coupled plasma emission spectrometry (ICP-ES), *in situ* Fourier transformed infrared (FTIR) spectroscopy, high-resolution transmission electron microscopy (HRTEM), spherical aberration-corrected scanning transmission electron microscopy with high-angle annular dark-field detector (Cs-corrected STEM-HAADF), X-ray photoelectron spectroscopy (XPS), and temperature programmed reduction of hydrogen (H_2_-TPR). In addition, we investigated the catalytic performance of Pd/TiO_2_ samples for selective hydrogenation by using phenylacetylene as a probe molecule. The photodeposited Pd/TiO_2_ sample with smaller Pd ensembles and more low coordination Pd sites exhibits enhanced styrene selectivity. Our results indicate that photodeposition may provide an alternative to preparation of supported Pd catalysts for achieving high alkene selectivity by controlling morphology and structure of metal nanoparticles.

## Results and Discussion

**Figure 1:**
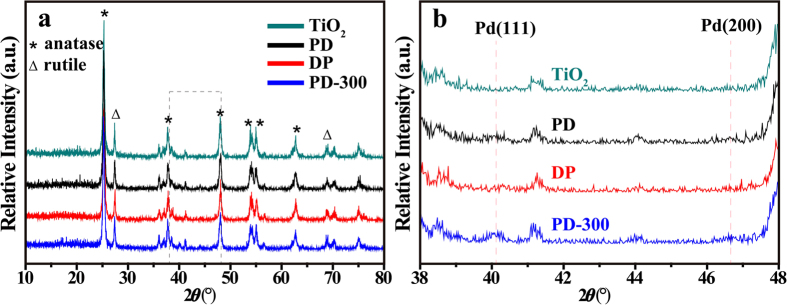
XRD patterns of samples of the TiO_2_ support, PD, DP, and PD-300 sample (**a**). (**b**) shows the magnifying XRD patterns from 2*θ* 38 to 48°.

XRD patterns of three Pd/TiO_2_ samples (PD, DP, and PD-300 prepared by photodeposition, deposition-precipitation and photodeposition with further thermal treatment at 300 °C in N_2_, respectively) and the TiO_2_ support are shown in Fig. 1. For the support sample, diffraction peaks with 25.2, 37.9, 48.6, 54.1, 55.3 and 62.7° are assigned to the (101), (004), (002), (105), (211) and (204) reflections of the anatase phase, while those of 27.4 and 68.7° are ascribed to the (110) and (301) reflections of the rutile phase^31^. Many researchers reported that if the particle size of Pd metal smaller than 4–5 nm, it is hard to discern the peaks of Pd (111) and (200) diffraction^32^,^33^. As shown in Fig. 1a, XRD patterns of PD, DP and PD-300 samples are similar to the TiO_2_ support and no characteristic diffraction for Pd can be identified, suggesting very small particle sizes (smaller than 5 nm) in these samples. The Fig. 1b shows that two new peaks with extremely weak intensity appear at 40.1 and 46.7° which correspond to the (111) and (200) planes of Pd (JCPDS 46–1043) indicating the presence of Pd metal species. Elemental analyses performed by using a ICP-ES measurement reveal that three Pd/TiO_2_ samples have the actual Pd loadings of about 1.0 wt%, which is in good agreement with the nominal values.

**Figure 2:**
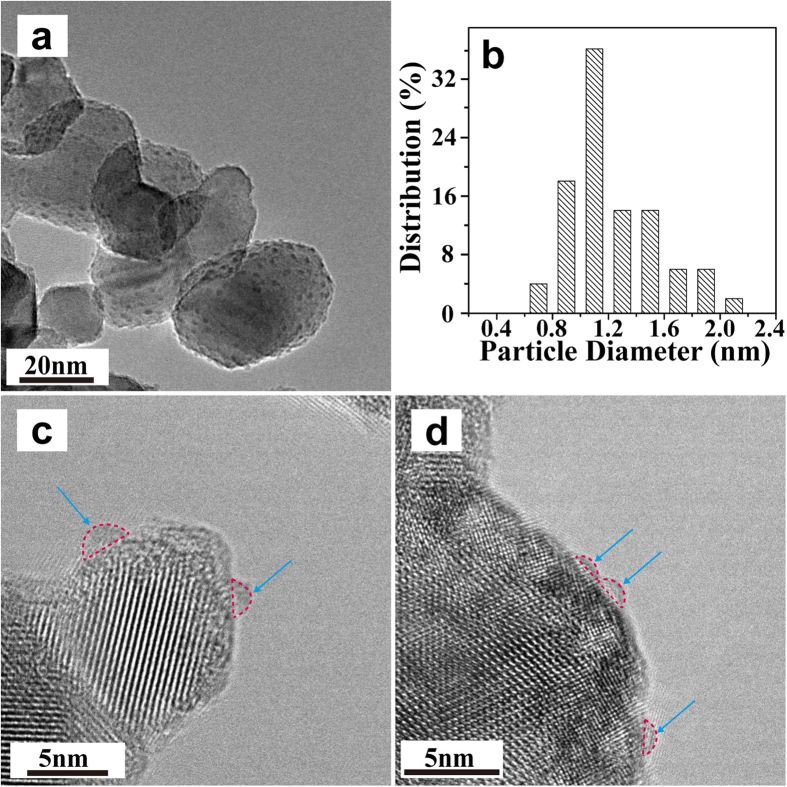
A HRTEM image (**a**) and the corresponding particle size distribution histogram (**b**) of the PD sample. (**c**) and (**d**) show the representative individual nanoparticles of (**a**). Pd particles are indicated by blue arrows.

**Figure 3:**
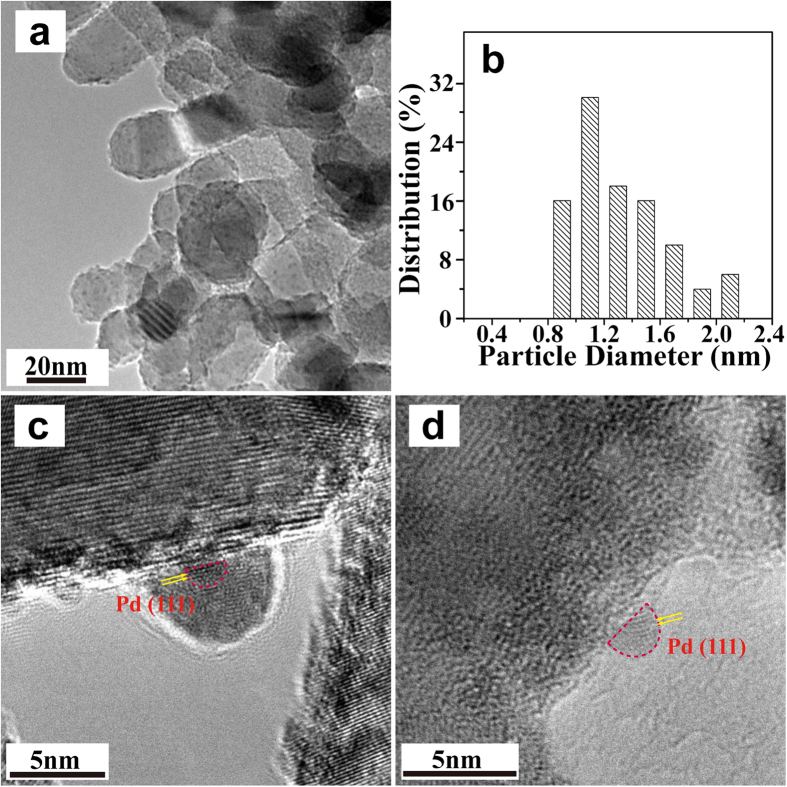
A HRTEM image (**a**) and the corresponding particle size distribution histogram (**b**) of the PD-300 sample. (**c**) and (**d**) show the representative individual nanoparticles of (**a**).

The HRTEM technique was used to observe the dispersion and morphology of Pd nanoparticles on the TiO_2_ support. For the PD sample, Pd nanoparticles are homogeneously distributed on the whole surface of the TiO_2_ support (Fig. 2a), with a narrow particle size distribution of 1–2 nm (Fig. 2b). Fig. 2c and d show the representative HRTEM images of some individual Pd nanoparticles. It is obvious that Pd nanoparticles lay prone on the TiO_2_ surface. The contact angle (CA) between the surface of the Pd nanoparticle and the outline of the metal-support interface is almost 65°, demonstrating the Pd nanoparticle has flattened sphere-like morphology. Moreover, it is hard to distinguish the particle boundaries of Pd nanoparticles in the TEM images. The disordered bulk structure of Pd particles on the TiO_2_ surface indicates that irregular arrangement of atoms constitutes the Pd metal particles. Ohyama *et al*.^23^ reported that Rh or Pt metal particles photodeposited on the TiO_2_ surface were not identified by TEM, although energy dispersive X-ray spectroscopy revealed that the metal species having a few dozen nanometers on the TiO_2_ surface. The authors suggested that the group VIII metal strongly interact with reducible metal oxide supports (*e.g.*, TiO_2_) when the supports are reduced, leading to the Rh or Pt metal (in our case, the Pd metal) a disordered structure on TiO_2_. In addition, for the PD-300 sample after high temperature treatment in N_2_, the size distribution of Pd particles is similar to the pristine PD sample, indicating a strong interaction between Pd particles and the support (Fig. 3a and b). It is worth noting that, the disorganized structure of Pd particles in the PD sample was changed by the thermal treatment. The PD-300 sample exhibits quasi-spherical morphology with obvious particle boundaries. The CA value of Pd particles increases to about 90° (Fig. 3c and d). Moreover, the spacing of crystallographic planes of 0.225 nm agrees well with the (111) lattice planes of Pd (Fig. 3c and d), indicating the rearrangement of Pd atoms and formation of crystalline structure under the 300 °C treatment in N_2_. As demonstrated by XRD results (Fig. 1b), two small Pd metal peaks around 40.1 and 46.7° appear for this sample.

**Figure 4:**
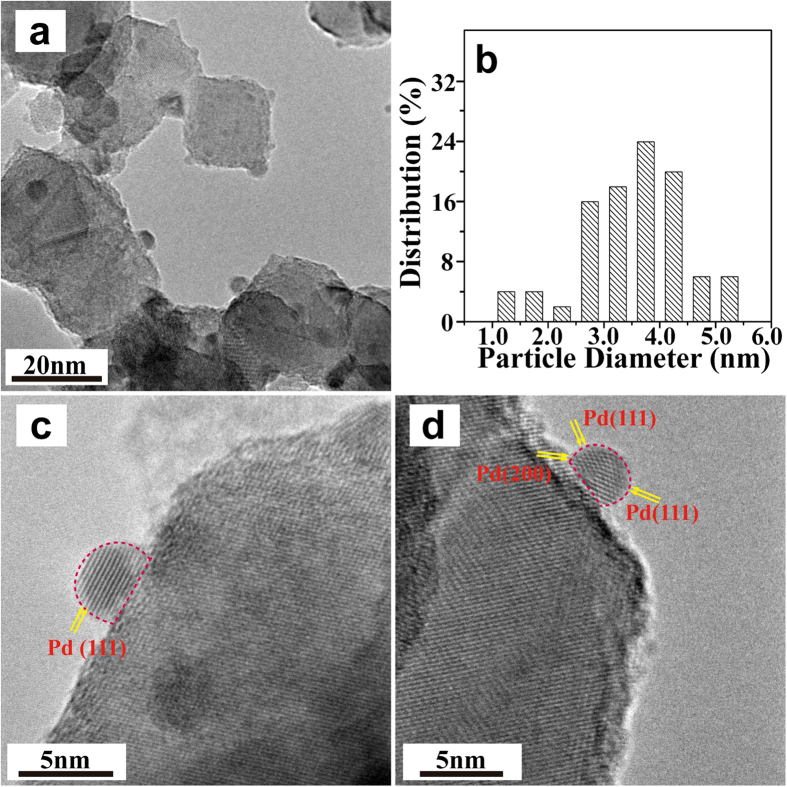
A HRTEM image (**a**) and the corresponding particle size distribution histogram (**b**) of the DP sample. (**c**) and (**d**) show the representative individual Pd nanoparticles.

For the DP sample, Pd nanoparticles with quasi-spherical morphology are deposited on the support showing wider particle size distribution and a larger average size about 3–5 nm (Fig. 4a and b). Pd atoms are clearly detected and regularly queued along the lattice planes in this sample (Fig. 4c and d). The lattice spacing of Pd particles at 0.225 and 0.195 nm can be determined by measuring the spaces between adjacent filamentary fringes, which could be ascribed to the (111) and (200) plane of Pd, respectively. Furthermore, well-organized crystalline surfaces and bulk structure of Pd metal particles with the CA value of about 110° can be obtained for the DP sample.

**Figure 5:**
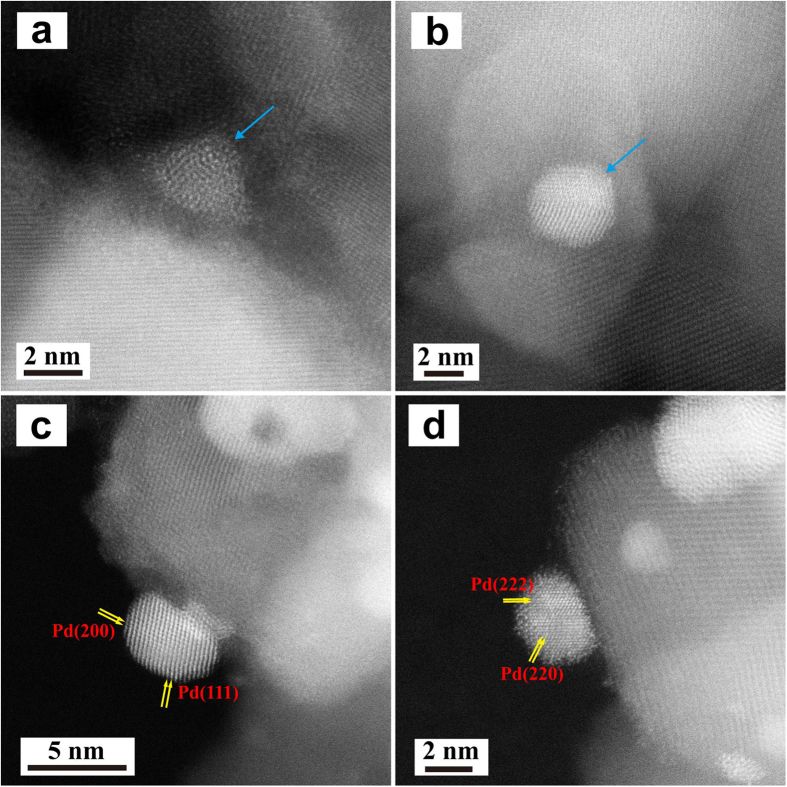
Representative Cs-corrected STEM-HAADF images of PD (**a**,**b**) and DP (**c**,**d**). Pd particles with low-ordered Pd atoms in the PD sample are indicated by blue arrows and panels of Pd particles with well-organized Pd atoms are shown in the DP sample.

The Cs-corrected STEM-HAADF technique (Fig. 5), which can provide directly interpretable atomic number (Z) contrast information, was utilized to further characterize the morphological and structural features of Pd nanoparticles supported on the TiO_2_ support by the PD and DP method. As shown in Fig. 5a and b, Pd particles in flattened spherical shape are located on the TiO_2_ surface and the interface of the Pd metal and TiO_2_ support is ambiguous. It is worth noting that the discontinuous phase of Pd metal in this sample containing the jumbled Pd atoms can be distinctly observed by using the atomic level STEM-HAADF, but it is difficult to discern the phase by the HRTEM (Fig. 2c and d) owing to the lack of a spherical aberration-corrector. By contrast, the bigger quasi-spherical Pd particles with well-organized structure and continuous phases are presented in the DP sample (Fig. 5c and d). The (200), (111), (222) and (220) lattice planes of face centered cubic Pd metal are distinct in the Cs-corrected STEM-HAADF images indicating Pd atoms are closely packed on the TiO_2_ surface during calcination and reduction and the particle boundaries, crystal facets, and metal-support interfaces are obvious. Liu *et al*.^30^ reported the formation of single Pd atoms on ethylene glycolate-stabilized ultrathin TiO_2_ nanosheets by the photodeposition. However, we did not observe the appearance of such single Pd atoms on the commercial anatase TiO_2_ support using the Cs-corrected STEM-HAADF.

**Figure 6:**
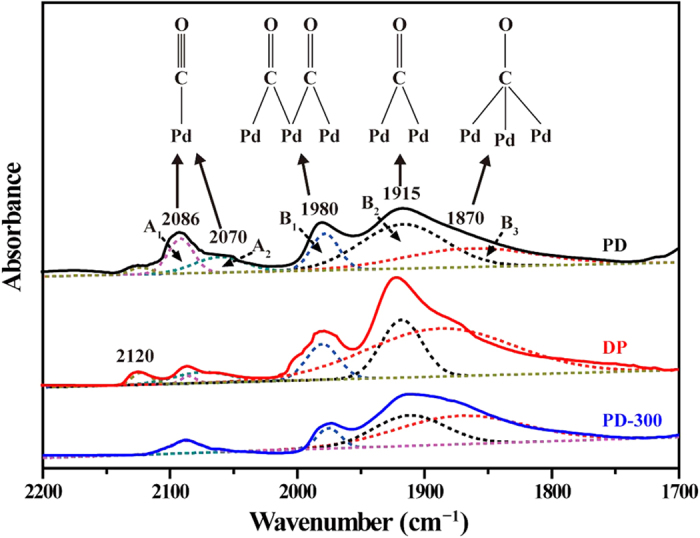
The deconvolution of CO-FTIR spectra of three catalyst samples.

Fig. 6 shows *in situ* FTIR spectra of CO adsorbed on three Pd/TiO_2_ samples. Analysis of FTIR spectra was carried out by a deconvolution method to calculate the relative value of different bond structures^34^. According to the different adsorption sites and intensity of CO on the Pd metal particles, the different surface information (such as steps, defects and terraces) in the supported Pd catalyst samples could be obtained. Two series of peaks (A- and B-type) can be distinguished roughly in the region of 2200–1800 cm^−1^, and all spectra were deconvoluted to elemental adsorption bands (Table 1): A_1_ (2070–2110 cm^−1^), A_2_ (2020–2070 cm^−1^), B_1_ (1950–2010 cm^−1^), B_2_ (1900–1940 cm^−1^), and B_3_ (1800–1890 cm^−1^). A_1_ and A_2_ peaks should be attributed to interrupted linear adsorption of CO on defects (steps, terraces and kinks) of Pd crystallites, or CO linearly adsorbed on well dispersed but not crystalline Pd^35^,^36^. Based on the density functional and vibrational spectroscopy studies, Yudanov *et al*.^37^ revealed that on-top adsorption on low-coordinated Pd centers (cluster corners and surface kinks) is stronger than that on regular (111) and (100) facets. The B_1_ peaks with a wavenumber range 1950–2010 cm^−1^ are suggested as bridge-bonded defective species, for example, bridged CO on surface steps or particle edges. Meanwhile, B_2_ and B_3_ peaks with the relatively low frequency range were assigned to CO bridge-bonded on (111) terraces or tri-coordinated CO adsorption^35^,^38^. It is noted that the presence of multiple CO adsorption (including bridged or threefold bonds) reflects the existence of the high coordination Pd sites in the Pd catalysts. For the DP sample, the B_1_, B_2_ and B_3_ bands shift toward higher wavenumbers compared with that of the PD sample, indicating the presence of more or less extended planes^35^. Meanwhile, the intensity of A_1_ and A_2_ bands at 2086 and 2070 cm^−1^ diminishes obviously. However, a strong band at 2120 cm^−1^ is observed, which may be attributed to the linear CO on Pd^+^ ions^39^. This result indicates that Pd nanoparticles of the DP sample are not totally reduced by H_2_. Fig. 6 also indicates the decrease of intensity of A_1_ and A_2_ bands for the PD-300 sample.

Based on the deconvolution of FTIR spectra, relative areas of linear bonds (A_1_ and A_2_ peaks) and multiple bonds (B_1_, B_2_ and B_3_ peaks) for three Pd/TiO_2_ samples are summarized in Table 1. The ratios of the relative area of linear bonds (R_A_) to the relative area of multiple bonds (R_B_) are also calculated and displayed in Table 1. The PD sample shows the relatively higher R_A_ and lower R_B_ value, with the R_A_/R_B_ ratio of 0.23 (approximating 0.07 for the DP sample). It demonstrates that Pd particles in the PD sample exhibit less ordered surfaces with more low coordination sites (surface defects, edges, steps, etc.)^38^. This conclusion is consistent with the above HRTEM and STEM-HAADF results that reveal that smaller Pd nanoparticles with disordered structure nature on the TiO_2_ surface can be obtained by the PD method. In addition, thermal treatment of the PD sample results in a decrease of R_A_/R_B_ from 0.23 to 0.08, demonstrating the reduction of defect sites and the change of structure of Pd particles will occur by transforming Pd atoms with low coordination into those with high coordination.

**Figure 7:**
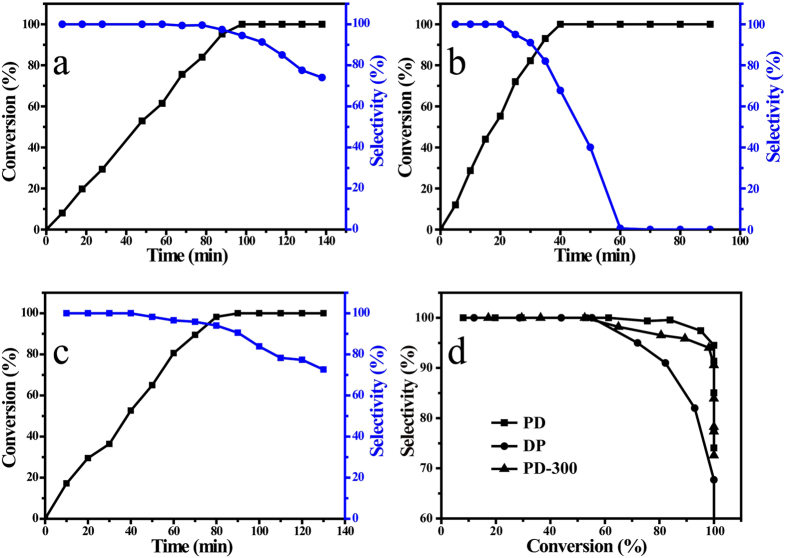
Selective hydrogenation of phenylacetylene to styrene in the presence of the PD (**a**), DP (**b**), and PD-300 (**c**) sample. Conversion of phenylacetylene vs selectivity to styrene is also presented as (**d**).

The heterogeneous hydrogenation of phenylacetylene was carried out in an autoclave or a batch reactor in previous researches^40^,^41^,^42^,^43^,^44^. The catalytic performance of the resulting Pd/TiO_2_ samples was evaluated by semihydrogenation of phenylacetylene to styrene. Fig. 7 shows the conversion of phenylacetylene and selectivity to styrene as a function of reaction time. For the PD catalyst sample, complete conversion of phenylacetylene occurred at 100 min (Fig. 7a). The DP catalyst sample exhibits higher initial activity, and the conversion of phenylacetylene reaches 100% at 40 min (Fig. 7b). The particle size may affect the specific activity of the Pd/TiO_2_ sample for phenylacetylene semihydrogenation. Some groups reported that the catalytic activity of supported Pd catalysts in selective hydrogenation of alkyne decreases as the Pd particles size decreases, especially when the average size is very small (≤3–5 nm), probably due to the different band structure characteristics of nano-sized metal compared to bulk metal^45^,^46^. The high activity for total hydrogenation of alkyne to alkane may result in low alkene selectivity. As shown in Fig. 7b, the selectivity to styrene for the DP sample drops straightly with the prolongation of reaction time. Especially, the selectivity falls quickly after the 100% conversion and 0% selectivity to styrene is obtained after 60 min. In contrast, the PD sample can keep high selectivity during the reaction: after reaction time 100 min, 95% selectivity is obtained (Fig. 7a). In addition, Fig. 7c exhibits that the PD-300 sample has a similar catalytic performance to the PD sample without thermal treatment, but the selectivity declines rapidly after the conversion reaches 100%. Fig. 7d presents the styrene selectivity as a function of phenylacetylene conversion, showing the different downward trends of the selectivity with the increase of conversion for the three samples. The selectivity to styrene in the same phenylacetylene conversion is in the order: PD > PD-300 > DP. It is worth noting that this order is consistent with the value of the R_A_/R_B_ ratio in Table 1 from the results of *in situ* FTIR spectra of CO adsorbed on three Pd/TiO_2_ samples.

**Figure 8:**
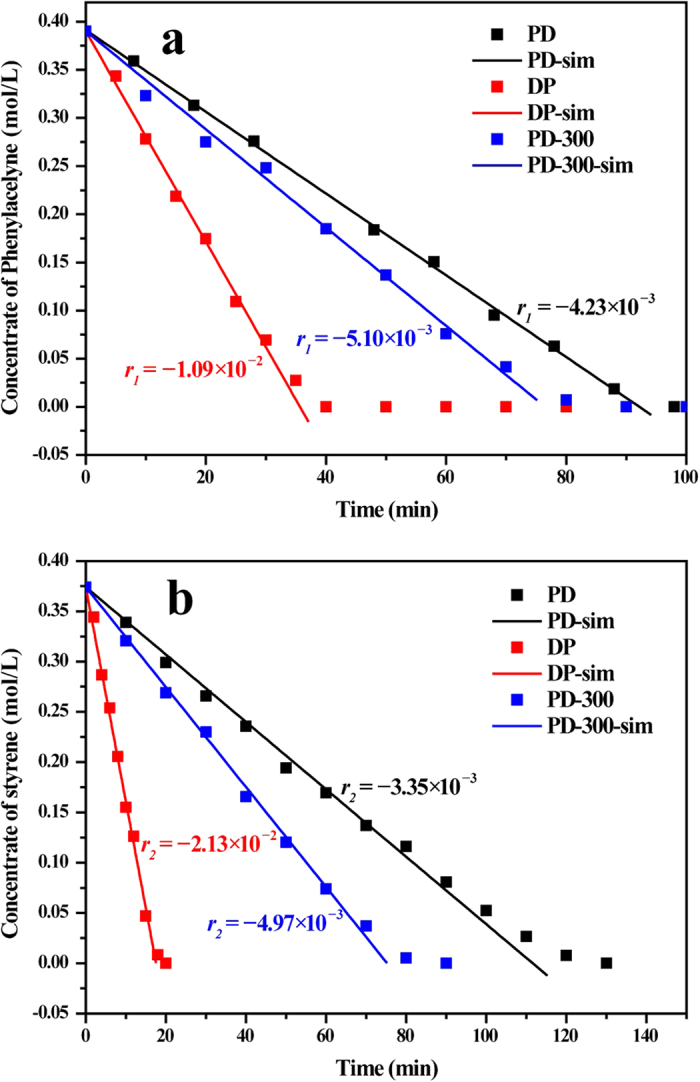
Concentration of phenylacetylene (**a**) and styrene (**b**) vs time during the phenylacetylene and styrene hydrogenation respectively. PD-sim, DP-sim and PD-300-sim lines are derived from the linear fit of data at the beginning of reactions to obtain approximate initial reaction rate *r*.

Detailed kinetic results such as initial reaction rate *r* and initial rate constant *k* in reactions from phenylacetylene to styrene and from styrene to ethylbenzene were computed to quantitatively analyze the influence of catalysts on the reaction process. Fig. 8a shows that the consumption of phenylacetylene with reaction time in hydrogenation of phenylacetylene to styrene, and the DP sample exhibits highest activity for the R_1_ reaction with an initial reaction rate −0.0109 mol L^−1^ min^−1^ from the slope of a fitted line. Compared with the DP sample, the PD sample catalyzed the R_1_ reaction with an only almost half reaction rate (−0.00423 mol L^−1^ min^−1^) and, after thermal treatment of the PD sample in 300 °C, the reaction rate increases to −0.00510 mol L^−1^ min^−1^. The similar trend among three samples is shown in Fig. 8b for hydrogenation of styrene to ethylbenzene R_2_. It should be noted that the DP catalyst with a reaction rate −0.0213 mol L^−1^ min^−1^ are over six times active than the PD sample with a reaction rate −0.00510 mol L^−1^ min^−1^ in hydrogenation of styrene. The value of reaction rate *r*, reaction rate constant k (calculated from Eqs (8) and (9)) and the ratio of *k*_*2*_/*k*_*1*_ are list in Table 2. The ratio of *k*_*2*_/*k*_*1*_ is used to quantitatively evaluate whether the catalyst facilitate styrene production and the lower ratio are more prone to maintain the higher styrene selectivity. The PD sample shows the lowest ratio of *k*_*2*_/*k*_*1*_ (0.792) indicating the PD sample may exhibit the highest selectivity of styrene in the semihydrogenation reaction of phenylacetylene due to the relative lower reaction rate in R_2_. Although the DP sample has both the highest *k*_*1*_ and *k*_*2*_, the *k*_*2*_ is great outweigh the *k*_*1*_. Therefore, this catalyst would have lowest selectivity of styrene because of the highest *k*_*2*_/*k*_*1*_ (1.954). In addition, The PD-300 sample derived from the PD sample after thermal treatment shows the ratio of *k*_*2*_/*k*_*1*_ increases from 0.792 to 0.975 indicating the decrease of selectivity to styrene during semihydrogenation. It should be noted that all results from kinetic analysis are consistent with results from catalytic evaluation of phenylacetylene semihydrogenation in which the PD sample have highest selectivity to styrene and the DP sample would generate more ethylbenzene at same phenylacetylene conversion. The kinetic parameters of this reaction show some difference between phenylacetylene semihydrogenation and acetylene semihydrogenation. Generally, the reaction constant of acetylene to ethylene is two or three orders of magnitude higher than that of ethylene to ethane^47^,^48^,^49^. However, the DP-catalyzed reaction exhibits the faster reaction of double bonds to single bonds, which would seem to be an anomaly in acetylene hydrogenation. From the previous research of phenylacetylene semihydrogenation, there is an order of magnitude difference between R_2_ and R_1_, and even the R_2_ shows the higher reaction constant than R_1_ in Pd-catalyst systems^50^,^51^,^52^. We suppose that the same catalysts show different reaction mechanisms between phenylacetylene semihydrogenation and acetylene semihydrogenation, mainly because the small molecular acetylene would dissolve in the subsurface of Pd metal to suppress formation of the β-hydride which extremely accelerates the reaction of ethylene to ethane and this process would not occur for the phenylacetylene, a relatively larger molecule^18^. So, the *k*_*2*_ > *k*_*1*_ would be normal in the phenylacetylene semihydrogenation.

**Figure 9:**
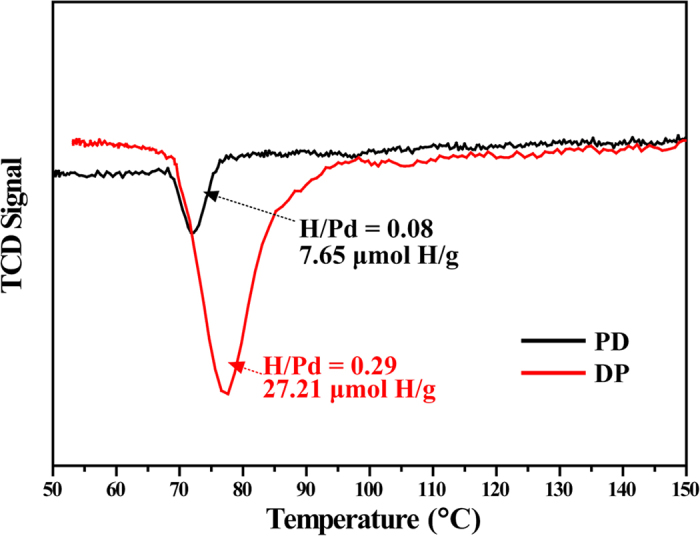
H_2_-TPR profiles of the PD and DP samples. The amounts of Pd per gram of the two Pd/TiO_2_ catalysts were calculated from results of the ICP experiment.

In order to elucidate the origin of the catalytic performance, we performed H_2_-TPR measurements for the PD and DP sample. As shown in Fig. 9, TPR profiles of both Pd catalysts show only one negative peak ranged from 67 to 95 °C. The negative peak centered in the region of 60–100 °C is attributed to the decomposition of hydrogen-saturated β-hydride^53^. It should be noted that the initiation of the decomposition peak is at the same temperature, 67 °C, indicating same activity of the β-hydride in two samples. However, the DP sample displays a higher peak intensity and a big peak area. The higher H/Pd value 0.29 demonstrates a large amount of β-hydride in this sample (H/Pd 0.08 for PD). Under hydrogenation conditions, unselective hydrogenation of alkyne proceeds on β-hydride which is feasibly formed on large Pd ensembles with high coordination Pd sites (terrace atoms), leading to total hydrogenation of alkyne to alkane and lowered alkene selectivity^7^,^8^. Thus, the larger Pd particles in the DP sample may generate more β-hydrides, which facilitate the total hydrogenation to alkane. According to literature, modifying morphology and structure of Pd particles for decreasing β-hydride phases may be beneficial for selective hydrogenation of phenylacetylene. For example, several groups have reported that the ethylene selectivity in acetylene hydrogenation over Pd catalysts is improved when the catalysts was modified with Si species either by selective deposition of Si on the Pd surfaces through chemical vapor deposition^11^ or by uniform distribution on the Pd surfaces through forming alloys with Pd^45^,^54^. The amounts of chemisorbed hydrogen are thus reduced on the Si-modified catalyst compared with those on the Pd-only catalyst mostly by geometrical dilution. On the other hand, Osswald *et al*.^16^ reported that the isolation of active Pd sites in crystallographic structure of Pd-Ga intermetallic compounds can result in decreasing availability of hydrogen because of the absence of Pd hydrides; Teschner *et al*.^18^ revealed that C dissolved in the top layers modifies the surface electronic structure of Pd and hinders the participation of subsurface H in the catalytic process. In our case, the above characterization results demonstrated that smaller Pd ensembles with low coordination Pd sites can be produced by the photodeposition method, which generate less β-hydrides in favor of selective hydrogenation of alkyne to alkene. Moreover, the value of R_A_/R_B_ ratios from the result of *in situ* FTIR has been used to estimate catalytic performance of Pd catalysts for selective hydrogenation of acetylene^7^. The disordered Pd metal blocking multiple adsorption of CO on high coordinated Pd sites could lead to the increase of the R_A_/R_B_ ratio, favoring high selectivity of acetylene to ethylene. Similarly, the higher styrene selectivity under the same phenylacetylene conversion is normally achieved at a higher R_A_/R_B_ ratio for the PD sample with more low coordination Pd sites.

**Figure 10:**
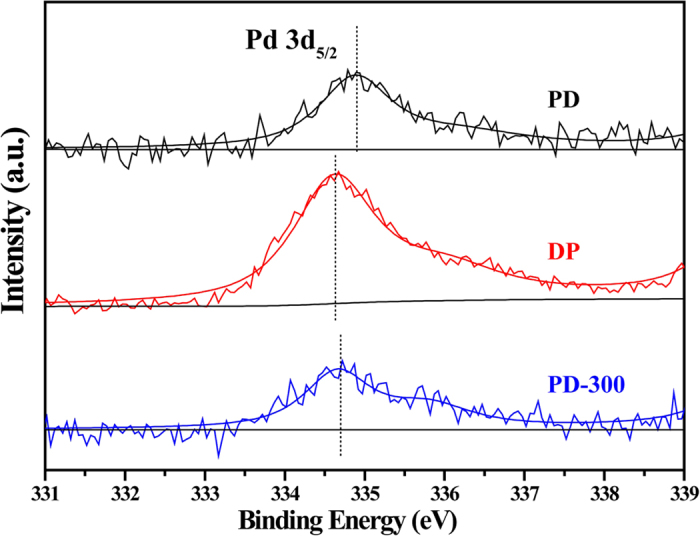
XPS spectra of Pd 3d_5/2_ for three Pd/TiO_2_ samples.

For exploring the possible electronic effect of the morphology and structure features of Pd particles, the surface information of three Pd/TiO_2_ samples were further evaluated using a XPS technique (Fig. 10). The binding energy (BE) of Pd 3d_5/2_ for the PD sample appears at 334.9 eV, which is of lower energy than that of a Pd foil at 335.2 eV^55^. The lower BE value indicates that the surfaces of Pd metal particles are more electron-rich than the Pd foil, thus the negative shift can be attributed to the electron donor property of the support TiO_2_. Wahlström *et al*.^56^ reported that photoirradiation to TiO_2_ generates TiO_2-*δ*_, which can serve as the formation site for metal particles. These TiO_2-*δ*_ species were suggested to contribute to an electron trap under the conduction band of TiO_2_, leading to a flow of excited electrons to TiO_2-*δ*_ and the reduction of metal ions on TiO_2-*δ*_^57^. Compared to that of the PD sample, DP and PD-300 samples have lower BE value of Pd 3d_5/2_ (334.6 and 334.7 eV, respectively), indicating that the surfaces of Pd metal particles of the PD sample has less electrons than that of the DP and PD-300 samples^58^. The more electron-deficient Pd in the PD sample could probably weaken the adsorption of product styrene on the Pd surface and inhibit the subsequent hydrogenation of styrene to ethylbenzene, resulting in high styrene selectivity. This result agrees with those of the PdCu bimetallic catalyst^9^ and the Pd/SiO_2_ catalyst with Pd silicide formation^45^, in which Pd is electron-deficient (larger binding energy from XPS results) compared to the monopalladium sample. In the latter case, the authors carried out additional hydrogenation reaction experiments with only styrene and the mixture of styrene and phenylacetylene as a reactant. The reaction results demonstrated that the commercial silica supported Pd catalyst (without Pd silicide) exhibited higher styrene conversion than the Pd/SiO_2_ with Pd silicide during hydrogenation of only styrene or the mixture of styrene and phenylacetylene^45^. Moreover, Fig. 6 shows that DP and PD-300 samples have lower BE value of Pd 3d_5/2_, compared with that of the PD sample, also indicating that the surface of Pd metal particles of the former two samples have more electrons and the stronger metal-support interaction^58^. In addition, the PD-300 sample also shows lower Binding Energy than the PD sample, demonstrating that the thermal treatment can make catalysts more surface electrons and stronger metal-support interaction. As the results from the (S)TEM, there is a strong correlation between the well-organized structure (in DP and PD-300 samples) and affluent surface electrons.

**Figure 11:**
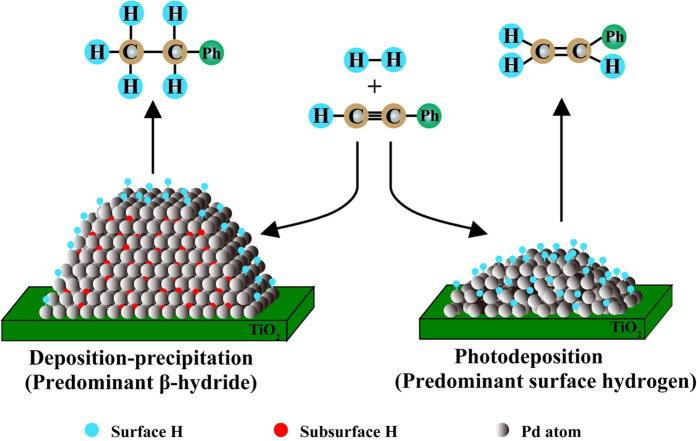
Schematic representation of Pd/TiO_2_ catalysts prepared by two methods for semihydrogenation of phenylacetylene to styrene. (For clarity, no phenylacetylene adsorbates are depicted on the surface; Ph refers to the phenyl group).

Schematic representation of the active sites of DP and PD catalyst samples which are responsible for total and selective hydrogenation of phenylacetylene is shown in Fig. 11. For the DP sample, large Pd ensembles are formed on the TiO_2_ support with regular arrangement of Pd atoms. A small amount of dissociated H atoms is adsorbed on the surface of Pd metal forming the surface H, which is responsible for selective hydrogenation of phenylacetylene to styrene. Meanwhile, dissolved subsurface H atoms are predominant in the Pd crystal lattices producing the β-hydrides and largely enhancing the total hydrogenation of phenylacetylene. By contrast, for the PD sample, the disordered structure may generate discontinuous spaces that enable H atoms permeating into the bulk of Pd metal and adsorbing around without formation of β-hydride phases. This unique structure of Pd particles is helpful in diminishing the hydrogen supply for the unwanted styrene hydrogenation. Therefore, the surface H atoms dissolved in the external and internal structure would be predominant in the PD sample, resulting in better selectivity to styrene due to more surface H atoms. On the other hand, the more electron-deficient Pd particles in the PD sample could probably weaken the adsorption of product styrene and inhibit the subsequent hydrogenation of styrene to ethylbenzene.

**Figure 12:**
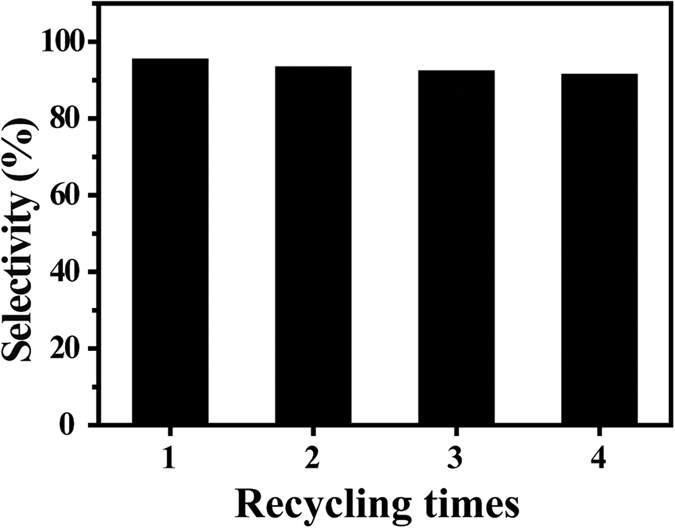
Reuse results of the PD sample for semihydrogenation of phenylacetylene to styrene. The conversion of phenylacetylene is 100%.

In addition, to check whether the catalyst can retain its hydrogenation selectivity, the PD sample was repeatedly used for the semihydrogenation of phenylacetylene for four times, and the experimental results are plotted in Fig. 12. The results show excellent reusability for this sample keeping high selectivity for the semihydrogenation.

In summary, the TiO_2_-supported Pd catalyst was fabricated by photodeposition for phenylacetylene semihydrogenation to obtain enhanced styrene selectivity. The effects of thermal treatment on the structural, morphological, and catalytic properties of the photodeposited Pd/TiO_2_ were also investigated. For comparison, a conventional deposition-precipitation method was used to prepare a contrastive Pd/TiO_2_ sample. HRTEM and Cs-corrected STEM-HAADF results demonstrate that flattened spherical Pd nanoparticles with ambiguous lattice fringes and indistinct particle boundaries were deposited on the TiO_2_ surface by the PD method. Larger quasi-spherical Pd ensembles are formed on the TiO_2_ support with regular arrangement of Pd atoms by the DP method. The disorganized structure of photodeposited Pd nanoparticles was changed by the thermal treatment at 300 °C exhibiting quasi-spherical morphology with obvious particle boundaries. Analysis of *in situ* FTIR spectra demonstrated that Pd particles in the PD sample exhibit less ordered surfaces with more low-coordination sites. The kinetic results show the rate constant ratio of styrene to phenylacetylene hydrogenation for the DP sample is much greater than the ratio for the PD sample suggesting the PD sample may obtain higher selectivity in phenylacetylene semihydrogenation. H_2_-TPR illustrated that the PD sample with disordered structure of Pd particles may prevent the formation of β-hydrides and probably produce more surface H atoms, which result in the excellent selectivity in phenylacetylene semihydrogenation. In addition, more metal surface electrons and stronger metal-support interaction in the DP and PD-300 samples, demonstrated by XPS, may suggest the well-organized structure from thermal treatment. The control of structural and morphological features of Pd nanoparticles by the photodeposition method imposes a beneficial effect on catalytic semihydrogenation selectivity.

## Methods

### Materials

Palladium chloride (PdCl_2_), phenylacetylene and sodium carbonate (Na_2_CO_3_) purchased from J&K Scientific Ltd. (Beijing, China) were of analytical grade and used without any further purification. Anatase TiO_2_ (purity > 99.9%) was obtained from Alfa Aesar Ltd. (Beijing, China). The deionized water with a conductance below 10^−6^ S cm^−1^ was used in all synthesis and washing processes.

### Catalyst preparation

Supported Pd catalysts with 1.0 wt% Pd contents were prepared by the photodeposition and deposition-precipitation method (denoted as PD and DP, respectively), with anatase TiO_2_ powder as the support and PdCl_2_ as the metal precursor compound. The PD catalyst was prepared as follows: TiO_2_ powder was mixed with distilled water containing a required amount of PdCl_2_, which was adjusted to yield catalysts containing 1.0 wt% Pd, followed by addition of isopropanol as the sacrificial agent under stirring. The suspension was then subjected to ultraviolet (UV) irradiation with a high-pressure Hg lamp (300 W) for 3 h. The sample was centrifuged and dried at 110 °C. The dried PD sample was further thermally treated at 300 °C in N_2_ (99.999%, 40 mL min^−1^) for 4 h, yielding the PD-300 catalyst.

For comparison, the conventional DP method was used to prepare supported catalysts with same Pd loadings as follows: anatase TiO_2_ was suspended in distilled water containing a required amount of PdCl_2_. Na_2_CO_3_ aqueous solution (0.25 mol L^−1^) was added dropwise to the suspension under stirring until pH value reached 10 and the suspension was constantly stirred for 5 h. The resulting suspension was then washed until no chloride anion was detected, and dried at 110 °C. Finally, the sample was calcined in a muffle furnace at 400 °C for 5 h and further reduced at 300 °C for 4 h in H_2_ (99.999%, 40 mL min^−1^), resulting the DP catalyst.

### Catalyst characterization

XRD patterns were recorded on a Shimadzu XRD-6000 diffractometer using Cu K*α* radiation (λ = 0.15406 nm) operated at 40 kV and 30 mA, with a scan step of 2*θ* 0.02° and a scan speed 10° min^−1^ in the 2*θ* range from 3° to 90°. The actual Pd loadings were analyzed by ICP-ES using a Shimadzu ICP-7500 instrument. HRTEM characterization was performed on JEOL JEM-2100F (200 kV). We counted the average size of 100 particles. Cs-corrected STEM-HAADF imaging was recorded using JEOL JEM-ARM200F (200 kV) equipped with a cold field emission gun and a spherical aberration corrector for probe correction. XPS was recorded using an ESCALAB250 X-ray photoelectron spectrometer equipped with monochromatized Mg K*α* X-ray radiation (1253.6 eV photons). The binding energy (BE) was determined by utilizing the C1s line as a reference with energy of 285.0 eV. *In situ* FTIR adsorption spectroscopy of CO experiments were recorded at resolution of 4 cm^−1^ on a Nicolet 380 instrument. The experiments were carried out in a custom-built quartz cell equipped with KBr windows allowing sample activation and successive measurements in the range of 20–550 °C. The Pd/TiO_2_ samples were pressed into a self-supporting wafer and placed in a quartz IR cell. After the cell was purged by N_2_ (99.999%, 50 mL min^−1^) at room temperature for 30 min, the catalyst was scanned to get a background record. Then the catalyst was exposed to a CO flow for 30 min and degassed by N_2_ (99.999%, 50 mL min^−1^) for 10 min to desorb the physical adsorption CO, and IR spectra were recorded. H_2_-TPR were conducted on Micromeritics ChemiSorb 2920, equipped with a thermal conductivity detector (TCD). The experiments consisted of exposure of 0.05 g Pd catalyst sample to a flow of 10% H_2_/Ar (50 mL min^−1^) at 50 °C for 30 min and then heating to 150 °C at 5 °C min^−1^.

### Evaluation of catalytic performance

Liquid-phase semihydrogenation of phenylacetylene and independent hydrogenation of styrene was carried out in a 250 mL stainless steel autoclave. 0.05 g of Pd/TiO_2_ catalyst was placed into the autoclave with 3 mL of phenylacetylene (styrene) and 67 mL of ethanol (as a solvent). After the autoclave was purged with 1.0 MPa hydrogen gas for three times, 0.5 MPa of H_2_ pressure remained. The reaction temperature was kept at 25 °C during the reaction. When the reaction time was reached, products could be obtained from a liquid outlet and were analyzed using a Shimadzu GC-2014C chromatographic instrument by means of a flame ionization detector and a 9006-PONA capillary column. The signals of phenylacetylene, styrene, and ethylbenzene (as the reactant, desirable product, and byproduct respectively in semihydrogenation of phenylacetylene) were recorded and n-heptane was used as a standard sample to analyze data. Catalytic activity, selectivity, concentration and the reaction rate were calculated, based on chromatographic data by the area normalization method, as follows:


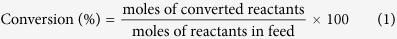



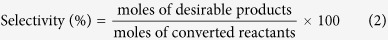



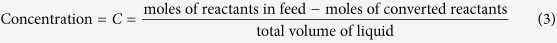



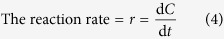


### Kinetic studies of hydrogenation

**Figure 13:**
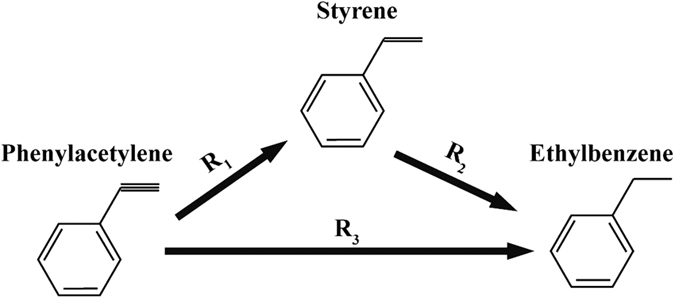
The scheme of phenylacetylene hydrogenation.

The Fig. 13 shows the processes of phenylacetylene hydrogenation. The calculation of kinetic results of phenylacetylene and independent styrene hydrogenation in the liquid phase was based on models from previous research^44^,^50^.


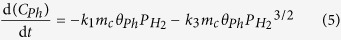



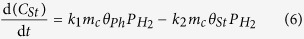



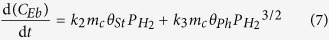


*C*_*i*_ = molar concentration of species *i*, mol/L^-1^

*t* = time, min

*k*_*i*_ = rate constant for reaction pathway *R*_*i*_, mol (Lg_cat_ MPa^1,3/2^ min)^-1^

m_c_ = catalyst mass, g

*θ* = surface concentration, mol/L^-1^

*PH*_*2*_ = pressure of hydrogen, MPa

*r* = reaction rate, mol L^−1^ min^−1^.

The R_3_ cannot occur in catalytic reaction based on the result of hydrogenation reaction. Along with initial condition, *θ*_*Ph*_ = 1, *θ*_*St*_ = 0 for independent phenylacetylene hydrogenation and *θ*_*Ph*_ = 0, *θ*_*St*_ = 1 for independent styrene hydrogenation, the transient reactor mass balance of Eqs (5,6,7) can be expressed as following equations:


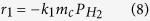


for the initial stage of phenylacetylene hydrogenation and


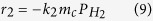


for the initial stage of independent styrene hydrogenation.

The *r*_*1*_ and *r*_*2*_ are initial rate of consumption of phenylacetylene and styrene. Therefore, in the initial condition, *r*_*1*_ and *r*_*2*_ exhibit a zero-order dependence on concentration of reactants along with results in the literature^43^,^50^.

## Additional Information

**How to cite this article:** Fan, Q. *et al*. Photodeposited Pd Nanoparticles with Disordered Structure for Phenylacetylene Semihydrogenation. *Sci. Rep.* **7**, 42172; doi: 10.1038/srep42172 (2017).

**Publisher's note:** Springer Nature remains neutral with regard to jurisdictional claims in published maps and institutional affiliations.

## Figures and Tables

**Table 1:** Relative areas of peaks from CO-FTIR for three catalyst samples.

Samples	Relative areas of linear bonds (R_A_)*		Relative areas of multiple bonds (R_B_)#			The ratio of R_A_/R_B_
	A_1_	A_2_	B_1_	B_2_	B_3_	
	2070–2110 cm^−1^	2020–2070 cm^−1^	1950–2010 cm^−1^	1900–1940 cm^−1^	1800–1890 cm^−1^	
PD	0.10	0.09	0.11	0.42	0.28	0.23
DP	0.01	0.05	0.10	0.21	0.63	0.07
PD-300	0.06	0.01	0.07	0.33	0.53	0.08

^*^The sum of relative areas of A_1_ and A_2_.

^#^The sum of relative areas of B_1_, B_2_ and B_3_.

**Table 2:** The initial reaction rate *r* and rate constant *k* of phenylacetylene and styrene hydrogenation for three catalyst samples.

	*r*_*1*_(mol L^−1^ min^−1^ 10^−3^)	*r*_*2*_(mol L^−1^ min^−1^ 10^−3^)	*k*_*1*_ (mol L^−1^ g_cat_^−1^ MPa^−1^ min^−1^)	*k*_*2*_(mol L^−1^ g_cat_^−1^ MPa^−1^ min^−1^)	*k*_*2*_/*k*_*1*_
PD	−4.23	−3.35	0.169	0.134	0.792
DP	−10.9	−21.3	0.436	0.852	1.954
PD-300	−5.10	−4.97	0.204	0.199	0.975
